# Circulating Soluble Neuropilin-1 in Patients with Early Cervical Cancer and Cervical Intraepithelial Neoplasia Can Be Used as a Valuable Diagnostic Biomarker

**DOI:** 10.1155/2015/506428

**Published:** 2015-03-19

**Authors:** Shouhua Yang, Henghui Cheng, Zaiju Huang, Xiaoling Wang, Yinglu Wan, Jing Cai, Zehua Wang

**Affiliations:** ^1^Department of Obstetrics and Gynecology, Union Hospital, Tongji Medical College, Huazhong University of Science and Technology, Wuhan 430022, China; ^2^Departments of Obstetrics, Gynecology, and Reproductive Sciences, Yale University School of Medicine, New Haven, CT 06520, USA; ^3^Institute of Pathology, Tongji Hospital, Tongji Medical College, Huazhong University of Science and Technology, Wuhan 430030, China

## Abstract

*Objective*. To investigate soluble neuropilin-1 (sNRP-1) in circulating and NRP-1 protein in cervical tissues from patients with cervical cancer or cervical intraepithelial neoplasia (CIN). *Methods*. sNRP-1 was measured in 64 preoperative patients and 20 controls. NRP-1 protein in cervical tissue was detected in 56 patients and 20 controls. *Results*. Both sNRP-1 and NRP-1 proteins were correlated with stage. sNRP-1 presented a high diagnostic ability of cervical cancer and CIN, with a sensitivity of 70.97% and a specificity of 73.68%. *Conclusions*. sNRP-1 in circulating can serve as a possible valuable diagnostic biomarker for cervical cancer and CIN.

## 1. Introduction

Cervical cancer is the fourth most common cancer in women worldwide, and it has the fourth highest mortality rate among cancers in women [1]. In developed countries, most cases of cervical cancer are preventable by routine screening and by treatment of precancerous lesion. But cervical cancer is still one of the leading causes of morbidity and mortality for women in most Asian and African areas that lack adequate protocols. Increased angiogenesis at the site of the primary tumor is associated with poor prognosis and relapse of cervical cancer [2, 3]. During carcinogenesis of cervical cancer, most blood vessel networks are generated through angiogenesis. Vascular endothelial growth factor (VEGF) is a key regulator of this process. Currently, VEGF and its receptors VEGFR-1, VEGFR-2, and Neuropilin-1 (NRP-1) are targeted in therapeutic strategies for vascular disease and cancer. As a receptor for VEGF, NRP-1 protein is reported to be upregulated in several cancers [4, 5], whereas the soluble NRP-1 (sNRP-1) is thought to act as an antagonist of signaling complex formation, which can inhibit the function of cell-associated NRP-1 [6]. However, the role of both sNRP-1 and NRP-1 proteins in patients with cervical cancer is still unclear. In this study, to delineate the roles of them in cervical cancer, the levels of sNRP-1 in circulation and NRP-1 protein in the cervical tissues of patients with cervical cancer and cervical intraepithelial neoplasia (CIN) were investigated, to clarify the clinical significance of them as well as the potential diagnostic implication for patients with cervical cancer.

## 2. Materials and Methods

### 2.1. Patients and Controls

All cases examined in this study were pooled from February 2012 to March 2014. In the 82 pathologically verified specimens of cervical tissue, there were 18 cases of CIN, 31 cases of squamous cancer (SCC), and 13 cases of adenocarcinoma (ACC) and 20 cases of benign disorders of uterus such as myoma or adenomyosis from the patients who underwent hysterectomy. Of the 44 invasive cases, there were 28-stage I a1 to I b1 (local early cervical cancer, LECC), and 16-stage I b2 to IIa (local advanced cervical cancer, LACC) based on classification criteria of the International Federation of Gynecology and Obstetrics (2000). All patients with malignant lesions underwent radical hysterectomy (or hysterectomy) and pelvic lymph node dissection, and 13 of them had pelvic lymph node metastasis. No patients were subjected to chemotherapy or radiotherapy before surgery. The age rank of the 82 patients was 24–66 years with mean of 44.7 years, and there were no significant differences among cervical cancer, CIN, and control groups. The serum samples were collected from all 82 patients, but the tissue samples were pooled from 74 patients only because 4 specimens of cervical cancer and another 4 specimens of CIN were lost. The sample collection procedure referred to the guidelines of the Ethics Committee of Union Hospital, Tongji Medical College, Huazhong University of Science and Technology, Wuhan, China.

### 2.2. Enzyme-Linked Immunosorbent Assay (ELISA)

The level of sNRP-1 in serum was assayed by a standardized sandwich enzyme-linked immunosorbent assay (ELISA) in triplicate according to the protocol recommended by the manufacturer. Blood samples were collected in the morning and centrifuged at 4°C to retrieve the serum. All serum samples were kept at −80°C until use. Concentrations of sNRP-1 were quantified by a human NRP-1 immunoassay (Cloud-clone Corp., Huston, Texas), and the detection range was from 0.313 to 20 ng/mL. Standards or samples were added to the appropriate microtiter plate wells with a biotin-conjugated antibody specific to NRP-1. Next, avidin conjugated to horseradish peroxidase (HRP) was added to each microplate well and incubated. After TMB substrate solution was added, only those wells that contain NRP-1, biotin-conjugated antibody, and enzyme-conjugated Avidin would exhibit a change in color. The enzyme-substrate reaction was terminated by the addition of sulphuric acid solution and the color change was measured spectrophotometrically at a wavelength of 450 nm ± 10 nm. The concentrations of sNRP-1 in the samples were then determined by comparing the O.D. of the samples to the standard curve.

### 2.3. Immunohistochemistry (IHC)

The expression of NRP-1 protein in cervical tissues from the cohort of 40 patients with cervical cancer and 14 patients with CIN, as well as 20 controls from normal cervix, were examined by IHC. The samples were fixed by formalin, embedded in paraffin, and cut into 5 *μ*m-thick sections. One paraffin block per subject was selected to be stained immunohistochemically with commercially available polyclonal antibody to NRP-1 (Abcam, Cambridge, MA). Sections were dewaxed in xylene and rehydrated with a concentration gradient of ethanol. After being blocked with 0.3% hydrogen peroxide, the antigen retrieval was carried out by a pressure cooker in citrate buffer (pH 6.0) at 121°C for 2-3 minutes. Slides were allowed to cool to room temperature for 20 minutes and then incubated in phosphate buffered saline (PBS) with 5% human serum for five minutes for blocking. The primary antibody (1 : 100) was applied at 4°C overnight. After washing with PBS three times, sections were incubated with a secondary anti-mouse horseradish peroxidase conjugated antibody for 30 minutes at room temperature and washed in PBS. The* staining* was developed by incubation with diaminobenzidine (DAB) solution for 15 minutes and sections were weakly counterstained with haematoxylin. Normal mouse IgG was substituted for primary antibody as the negative control. In each section, four high-power visual fields were selected and observed. 500 atypical cells or normal cervical cells were counted in each sample. The score of intensity: weak or no staining received a score of 1, moderate staining received a score of 2, strong staining received a score of 3, and results are scored by multiplying the percentage of positive cells (*P*) by the intensity (*I*). The formula is *Q* = *P* × *I*, maximum = 300.

### 2.4. Statistical Analysis

Continuous variables were expressed as mean ± SD; the data from each category was presented as frequency and percentage. Linear regression was used to analyze the ELISA data, serum levels of circulating sNRP-1 were tested for correlation with patients' stage of disease, pathological types, and metastasis with independent sample *t*-test and one ANOVA. ROC curve was used to analyze the diagnostic value of sNRP-1. Fisher's exact test was used to analyze the incidence of NRP-1 protein between different groups. Correlation between sNRP-1 level and NRP-1 protein was analyzed by Spearman's correlation and coefficient. Analysis was performed using SAS 6.12 (SAS Institute, Cary, NC) or SPSS 12.0 on a Windows computer. A minimum *P* < 0.05 was considered to be statistically significant.

## 3. Results

### 3.1. Elevated Levels of Circulating sNRP-1 in Patients with Cervical Cancer and CIN

**Figure 1:**
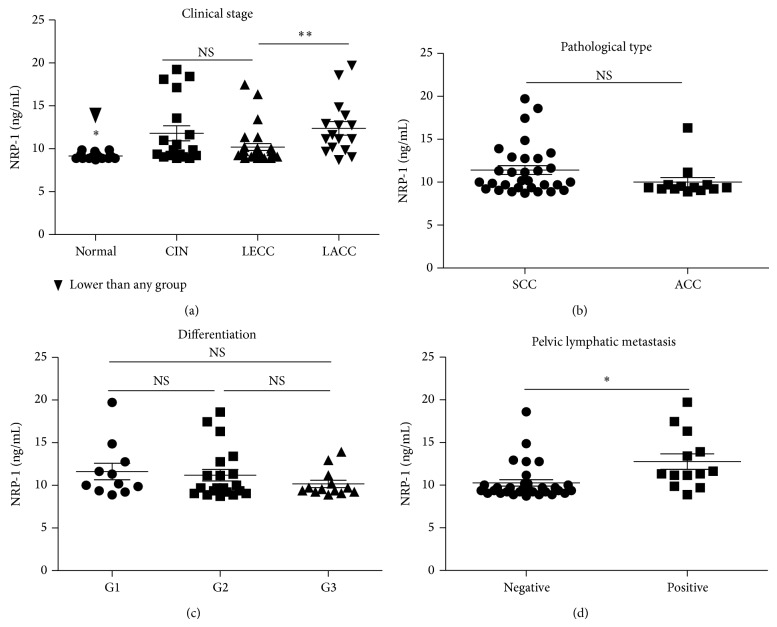
The detection of circulating sNRP-1 in patients with cervical cancer and CIN. (a) The sNRP-1 levels of CIN and cervical cancer groups were much higher than those of the control group; (b) there was no significant difference in circulating sNRP-1 between SCC and ACC groups; (c) among well, moderate, and poor tumour cell differentiation groups, there were no obvious differences of sNRP-1; (d) the sNRP-1 levels in patients with positive pelvic lymphatic metastasis were much higher than those of the negative group.

The serum samples were tested with ELISA to determine the levels of circulating sNRP-1 in the blood, as described in section of Materials and Methods. We found that circulating sNRP-1 levels in patients with cervical cancer (mean, 10.99768143 ng/mL; median, 9.696935 ng/mL; range, 8.726887 to 19.72077 ng/mL) and CIN (mean, 11.78970578 ng/mL; median, 9.858609 ng/mL; range, 8.888561 to 19.23574 ng/mL) were significantly higher than those of the controls (mean, 9.160855 ng/mL; median, 9.050236 ng/mL; range, 8.726887 to 9.858609 ng/mL) (*P* < 0.01, resp., Figure 1(a)). However, there was no significant difference between sNRP-1 levels of CIN and cervical cancer groups (*P* > 0.05), while the sNRP-1 level of LECC was much lower than LACC group (*P* < 0.01, Figure 1(a)). The sNRP-1 of LECC was still higher than control group (*P* < 0.05).

As described before, we also subdivided cervical cancer group based on the pathological types, pelvic lymphatic node status, and cell differentiation. The sNRP-1 level in patients with positive pelvic lymphatic metastasis was much higher than that of the negative group ((12.76 ± 0.9070) ng/mL versus (10.26 ± 0.3793) ng/mL, *P* < 0.01, Figure 1(d)). There was no significant difference between SCC and ACC groups ((11.41 ± 0.5228) ng/mL versus (10.01 ± 0.5484) ng/mL, *P* = 0.1198, Figure 1(b)). Among three different cell differentiation groups, there were no significant differences between any two groups (*P* > 0.05, resp., Figure 1(c)).

### 3.2. Circulating sNRP-1 as Diagnostic Biomarker of Invasive and Precancerous Cervical Disorders

**Figure 2:**
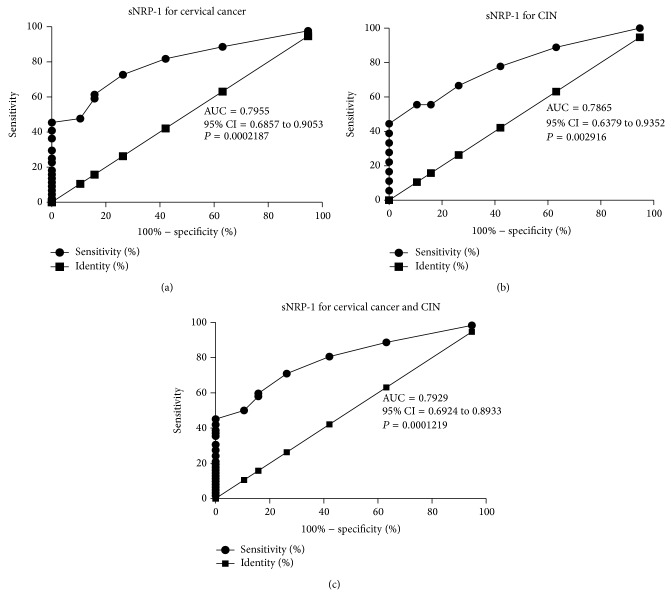
Soluble NRP-1 in circulation can be served as a valuable diagnostic biomarker of cervical cancer and CIN by ROC analysis.

To test whether the sNRP-1 based biomarker could distinguish cervical cancer and CIN from controls and to figure out its diagnostic value for invasive and precancerous cervical disorders, ROC curves analysis was constructed between benign controls and cervical precancerous or (and) invasive diseases. The AUC of using sNRP-1 as diagnostic biomarker for cervical cancer was 0.7955 (95% CI = 0.6857 to 0.9053; *P* = 0.0002187) (Figure 2(a)). For CIN was 0.7865 (95% CI = 0.6379 to 0.9352; *P* = 0.002916) (Figure 2(b)). For cervical cancer and CIN together, the AUC was 0.7929 (95% CI = 0.6924 to 0.8933, *P* = 0.0001219) (Figure 2(c)), indicating the feasibility of sNRP-1 to diagnose both cervical cancer and CIN. Sensitivity, specificity, and all cutoff values of sNRP-1 levels were determined using ROC analysis. Taking cervical cancer and CIN together, the sNRP-1 cutoff value of 8.808, 8.969, 9.131, 9.293, 9.454, 9.616, and 9.778 ng/mL yielded sensitivities of 98.39, 88.71, 80.65, 70.97, 59.68, 58.06, and 50% and specificities of 5.263, 36.84, 57.89, 73.68, 84.21, 84.21, and 89.47%, respectively. Based on this data, a level of 9.293 ng/mL (the sum of sensitivity and specificity was the highest) was determined to be the most efficient threshold, so we set this level as the cutoff value. The sensitivity of the assay was 70.97%, the specificity was 73.68%, and the likelihood ratio was 2.70.

### 3.3. The Expression of NRP-1 Protein in Tissues of Uterine Cervix

**Figure 3:**
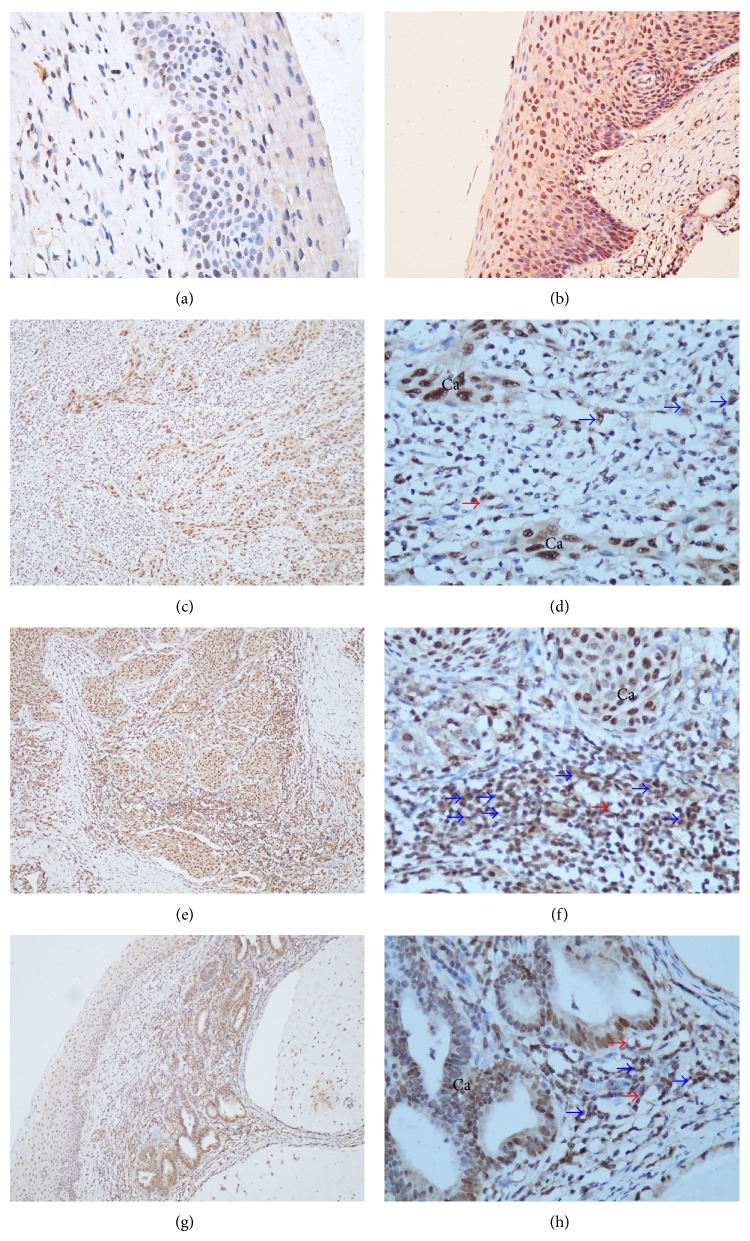
The expression of NRP-1 protein in invasive and precancerous cervical tissues. (a and b) Expression of NRP-1 in CIN tissues ×200; (c–f) expression of NRP-1 protein in SCC tissue, red arrows point to endothelial cells and blue arrows point to immune cells. (c and e) ×100, (d and f) ×400; (g and h) expression of NRP-1 protein in ACC tissue, red arrows point to endothelial cells and blue arrows point to immune cells. (g) ×100, (h) ×400.

The positive staining of uneven brown-yellow granules of NRP-1 protein was mainly located in cytoplasm and partly in membrane of heteromorphic cells and endothelial cells. A few stained cells were scattered in the section, and the morphological identification showed that these cells were from immune system (Figure 3). The expression of NRP-1 protein was significantly increased in both cervical cancer and CIN groups compared with control group (*P* < 0.01, resp.), but there were no significant differences among CIN, LECC, and LACC groups, and there was no significant relationship between the positive rate and pathological types or pelvic nodal statuses (*P* > 0.05, resp.). However, the occurrence of NRP-1 protein positive granules was lower in cells of the poor tumor cell differentiation group than both moderate and well tumor cell differentiation groups (*P* < 0.05, resp.); the detail was shown in Table 1.

### 3.4. The Correlation between sNRP-1 and Histochemical NRP-1 in Cervical Cancer and CIN

**Figure 4:**
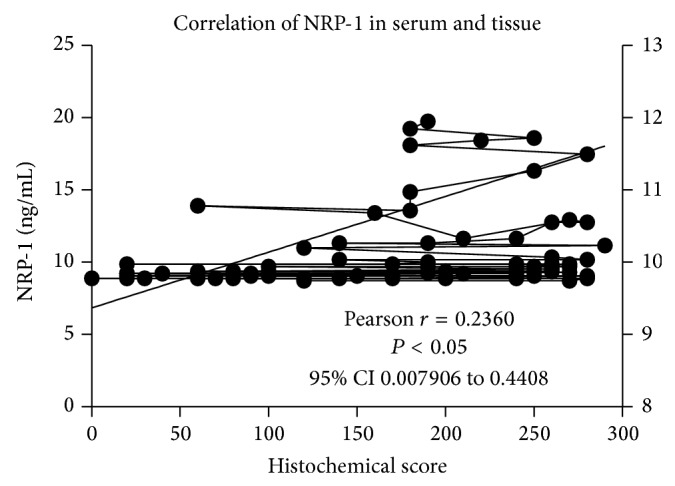
The correlation of soluble NRP-1 in circulation and NRP-1 protein in cervical tissues from the same patients.

We collected samples of both serum and cervical tissue from 74 patients. Although there was a statistical difference between serum and protein levels, NRP-1 protein expression in cervical tissues correlated with sNRP-1 level in circulation from each patient. The correlation coefficient of serum NRP-1 and uterine cervix tissue NRP-1 was 0.2360 in Spearman test and *P* value was 0.0429 and the 95% CI 0.007906 to 0.4408 (Figure 4).

## 4. Discussion

The growth of new blood vessels is needed for solid tumors to grow and metastasize, and the molecular mechanisms underlying angiognesis have become increasingly clear. The VEGF family is an essential player in this process [4, 7]. VEGF mainly operates by interacting with three receptors: VEGFR-1, VEGFR-2, and NRP-1. Although these receptors are expressed in spatially and temporally overlapping patterns, they are thought to play different roles in VEGF signaling [8]. NRP-1 is a multifaceted transmembrane receptor that not only binds VEGF and forms a complex with VEGFR-2 but also binds a structurally and functionally unrelated family of traditional axon guidance cues SEMA-3. Compared to the main receptors of VEGF, NRP-1 function in conjunction with multiple ligands and receptors to guide vascular development remains elusive [5, 9]. NRP-1 regulates endothelial cell adhesion to extracellular matrix proteins independently of VEGFR-2. Based on its dual role as an enhancer of VEGF activity and a mediator of endothelial cell adhesion, NRP-1 also emerges as a promising molecular target for the development of antiangiogenic drugs. Endothelial cells express NRP-1, and NRP-1 associates with the receptor tyrosine kinase VEGFR-2 after binding VEGF-A to enhance angiogenesis [10, 11]. Nevertheless, sNRP-1 was thought to be an antagonist of NRP-1, which can inhibit the function of NRP-1 protein [6].

NRP-1 overexpression has been reported in a variety of human cancers, including those derived from carcinomas of the prostate, kidney, bladder, stomach, colon, pancreas, breast, ovary, lung, liver, nasopharynx, and brain [5, 12–17]. In most solid tumors, high expression of NRP-1 is significantly correlated with clinical stage, angiogenesis, node invasion, and poor overall survival [5, 13, 14, 16, 18]. Analyzing NRP-1 protein could be used to predict the shorter overall survival and relapse-free survival rate and related to the complete remission response in acute myeloid leukemia. Therefore, NRP-1 may also act as a more aggressive and promising predictor for the poor prognosis of acute myeloid leukemia [19–21]. Besides NRP-1 protein, soluble NRP-1 in circulation has also been identified in human serum by both ELISA and western blot before [22]. They described mean sNRP-1 levels of (322 ± 82) ng/mL with a range of 146–618 in normal individuals. In our study the mean sNRP-1 level was only 9.161 ± 0.07726 ng/mL with a range of 8.726887–9.858609 ng/mL in normal control groups. We repeated the experiment three times with a series of standard samples each time. The main reason for the difference between this study and the previous research is most likely the result of a different ELISA system because the ELISA Kits and standard samples were purchased from the different companies.

In this study, we first verified the increased level of sNRP-1 in circulation and the focal expression of NRP-1 protein in a subset of cervical cancerous and precancerous patients by ELISA and IHC, respectively. Our results showed that, compared to controls, sNRP-1 and NRP-1 protein levels were frequently upregulated in samples from both cervical cancer and CIN. In contrast, the sNRP-1 levels were much lower in normal controls, and only a few populations of normal cervical tissues showed positive staining for NRP-1. Next, for both sNRP-1 and cell-associated NRP-1, there were no significant differences between cervical cancer and CIN groups, suggesting that the upregulation of both molecules was an early event during the carcinogenesis of cervical cancer. Further analysis revealed that high sNRP-1 level was correlated closely with tumor stage and pelvic lymphatic nodal metastasis. These findings implicated that sNRP-1 may also be related to overall survival.

Importantly, as evidenced by the ROC analysis, the level of circulating sNRP-1 was a valuable diagnostic biomarker of cervical cancer and CIN. Although conventional clinicopathologic parameters such as grade, stage, and lymphatic node status of the tumor are widely considered to be predictors of metastasis, recurrence, and patients survival for cervical cancer, gynecologists still intend to explore novel biomarkers associated with the histopathological features and biological behavior of this disease. These biomarkers may be more efficient for early diagnosis, enabling the predication of the carcinogenesis of precancerous lesions and metastasis before positive image discovery. In this study, we found that sNRP-1 level in circulation was significantly increased after the imitation of the CIN stage, and it also could be used as a biomarker for cervical progression and a novel target for therapeutic antibody engineering human cervical cancer treatment. We also have tried to analyze the prognosis of the patients involved in this study, but we found only two recurrent cases and no death during the past 6–30 months as the cases in this study were all early stages, and it is difficult to figure out the relation between NRP-1 and the prognosis of early cervical cancer.

The overexpression of NRP-1 has been shown to regulate not only angiogenesis, but also other aspects of tumorigenesis, such as modulation of apoptosis and cell migration in human colon cancer [23], epithelial-mesenchymal transition of human ovarian cancer, [24] and especially tumour-induced immune tolerance [25]. In cervical cancer, the positive NRP-1 staining can be found in cancer cells, endothelial cells, and immune cells (Figure 3), and the role of these immune cells in cervical cancer is still unclear. NRP-1 protein is upregulated on tumor-infiltrating lymphocytes (TILs) and can be induced on peripheral blood mononuclear cells by tumor tissue [26]. As a therapeutic target, NRP-1 may allow selective targeting of TILs subsets including suppressive Tregs [26]. The stability and function of Tregs are maintained by a neuropilin-1-semaphorin-4a axis. This pathway is a potential therapeutic target that could limit Treg-cell-mediated tumour-induced tolerance without inducing autoimmunity [25]. Gene deletion of NRP-1 in macrophages favors TAMs' entrapment in normoxic tumor regions, which abates their proangiogenic and immunosuppressive functions and inhibits tumor growth and metastasis [27]. This phenomenon suggests that the NRP-1 may play a special role in the tumor-infiltrating immune cells of cervical cancer.

sNRP-1 and cell-associated NRP-1 have opposite functions (inhibitor and enhancer of angiogenesis, resp.) and they come from alternative splicing [6]. In this study we found that there was a weak (Pearson *r* = 0.2360) but still significant (*P* = 0.0429) correlation between the sNPR-1 and NRP-1 protein of cervical tissues from the same women. This may implicate sNRP-1 responses to NRP-1 protein in cervical cancer and CIN. This is an interesting hypothesis but still needs to be verified in the future, and it may become a new therapeutic agent of advanced cervical cancer.

## 5. Conclusion

In summary, this study demonstrated that the levels of circulating sNRP-1 and cell-associated NRP-1 protein were highly increased in patients with CIN and cervical cancer. These results suggested that overexpression of NRP-1 may play an important role in the carciongenesis of cervical cancer and emerge as a promising molecular target for the cervical carcinogenesis. Importantly, the level of circulating sNRP-1 can be used as a possible valuable diagnostic biomarker of both CIN and cervical cancer.

## Figures and Tables

**Table 1:** Expression of NRP-1 protein in 74 cervical tissues.

Factor	Classification	*n*	NRP-1 positive (%)	*P* ^*^
Age	>40 y	33	23 (69.70)	>0.05
	⩽40 y	7	6 (85.71)	

Clinical stage	Controls^*^	20	4 (20.0)	<0.01
	CIN	14	10 (71.43)	
	LECC	25	18 (72.0)	
	LACC	15	11 (73.33)	

Nodal metastasis^**^	Absent	28	21 (75.0)	>0.05
	Present	12	8 (66.67)	

Pathological grade	HG1	7	5 (71.43)	0.037
	HG2	25	21 (84.0)	
	HG3	8	3 (37.5)	

Pathological types	SCC	31	22 (70.97)	>0.05
	ACC	9	7 (77.77)	

SCC: squamous cancer, ACC: adenocarcinoma, CIN: cervical intraepithelial neoplasia, LECC: local early cervical cancer, stage I a1 to I b1, and LACC: local advanced cervical cancer, stage I b2 to IIa. ^*^The occurrence of NRP-1 protein of this group was much lower than that of the other 3 groups. ^**^Pelvic lymphatic nodes involved only.
